# Intermittent compressive force induces cell cycling and reduces apoptosis in embryoid bodies of mouse induced pluripotent stem cells

**DOI:** 10.1038/s41368-021-00151-3

**Published:** 2022-01-04

**Authors:** Jeeranan Manokawinchoke, Phoonsuk Limraksasin, Hiroko Okawa, Prasit Pavasant, Hiroshi Egusa, Thanaphum Osathanon

**Affiliations:** 1 Division of Molecular and Regenerative Prosthodontics, Tohoku University Graduate School of Dentistry, Sendai, Miyagi 980-8575 Japan; 2 Dental Stem Cell Biology Research Unit and Department of Anatomy, Faculty of Dentistry, Chulalongkorn University, Bangkok, 10330 Thailand; 3 Center for Advanced Stem Cell and Regenerative Research, Tohoku University Graduate School of Dentistry, Sendai, Miyagi 980-8575 Japan

**Keywords:** Stem-cell differentiation, Stress signalling

## Abstract

In vitro manipulation of induced pluripotent stem cells (iPSCs) by environmental factors is of great interest for three-dimensional (3D) tissue/organ induction. The effects of mechanical force depend on many factors, including force and cell type. However, information on such effects in iPSCs is lacking. The aim of this study was to identify a molecular mechanism in iPSCs responding to intermittent compressive force (ICF) by analyzing the global gene expression profile. Embryoid bodies of mouse iPSCs, attached on a tissue culture plate in 3D form, were subjected to ICF in serum-free culture medium for 24 h. Gene ontology analyses for RNA sequencing data demonstrated that genes differentially regulated by ICF were mainly associated with metabolic processes, membrane and protein binding. Topology-based analysis demonstrated that ICF induced genes in cell cycle categories and downregulated genes associated with metabolic processes. The Kyoto Encyclopedia of Genes and Genomes database revealed differentially regulated genes related to the p53 signaling pathway and cell cycle. qPCR analysis demonstrated significant upregulation of *Ccnd1*, *Cdk6* and *Ccng1*. Flow cytometry showed that ICF induced cell cycle and proliferation, while reducing the number of apoptotic cells. ICF also upregulated transforming growth factor β1 (Tgfb1) at both mRNA and protein levels, and pretreatment with a TGF-β inhibitor (SB431542) prior to ICF abolished ICF-induced *Ccnd1* and *Cdk6* expression. Taken together, these findings show that TGF-β signaling in iPSCs enhances proliferation and decreases apoptosis in response to ICF, that could give rise to an efficient protocol to manipulate iPSCs for organoid fabrication.

## Introduction

Induced pluripotent stem cells (iPSCs), which can be generated by reprogramming of somatic cells including oral tissue cells,^1^ possess unlimited self-renewal property and can differentiate into any type of cell and tissue. Therefore, iPSCs are considered to be a promising tool not only for tissue regeneration but also for disease modeling by in vitro fabrication of three dimensional (3D) tissues/organs (organoids).^2^ Recently, technologies for engineered cell manipulation have been actively investigated to control 3D cell-cell interactions of stem cells to generate organoids. Mechanical stress is a promising manipulation technique for organoid formation of iPSCs.^3^

Mechanical force regulates numerous biological responses in stem cells.^4^ Mechanical stimulation induces differentiation and maturation of iPSCs toward bone and cartilage cell lineages.^5,6^ Application of shear stress to pluripotent embryonic stem cells (ESCs) promotes endothelial cell and hematopoietic cell differentiation.^7^ In contrast, specific hydrodynamic forces in 3D shaking culture maintain and restore multipotency of mesenchymal stem cell (MSC) spheroids.^8^ Low-intensity vibration attenuates adipogenic differentiation in MSCs.^9^ Thus, the effects of mechanical force on stem cell responses substantially depend on many factors related to both force and cells, including force type, force magnitude, treatment duration, cell type, and cell stage.

In the field of oral science, intermittent compressive force (ICF) has been particularly well investigated with regard to the regulation of cell behaviors in association with mastication, biting or orthodontic treatments. ICF stimulates osteogenic differentiation in human periodontal ligament stem cells (PDLCs) via the transforming growth factor (TGF-β) pathway.^10^ In human bone-derived cells, ICF activates the Wnt pathway and subsequently promotes osteogenic differentiation.^11^ Expression of receptor activator of nuclear factor κB ligand (RANKL) is increased under ICF application via regulation of interleukin 1β in PDLCs.^12^ In mouse pre-osteoblasts, ICF upregulates Notch target gene expression through the TGF-β pathway.^13^ This evidence suggests that ICF regulates many signaling pathways and further influences a variety of cell responses; therefore, a systematic analysis is required to investigate how ICF affects cell responses.

Global gene expression profiles have been investigated in various conditions to determine the potential pathways regulated by mechanical force. ICF-treated PDLCs show a significant change of gene expression in focal adhesion, regulation of actin cytoskeleton, TGF-β signaling, and cytokine-cytokine receptor pathways.^10,14^ PDLCs treated with orthodontic force exhibit changes in cell cycle, DNA replication, immune system, and metabolism pathways.^15^ Cyclic stretch treatment in embryonic mouse cardiomyocytes leads to significant changes in gene ontology categories related to contractile fiber parts, myofibrils, contractile fibers, and regulation of cellular component organization.^16^ In this context, the development of effective regenerative medicine and engineered cell manipulation approaches requires understanding of global gene expression profiles in response to mechanical stimulation, particularly in iPSCs, which are capable of sophisticated organoid formation in vitro. However, the effect of ICF on iPSCs remains unknown. The present study aimed to identify the ICF-regulated pathways in 3D-cultured mouse iPSCs by global gene expression analysis using RNA sequencing.

## Results

### Differential gene expression profile in ICF-treated iPSCs

**Fig. 1:**
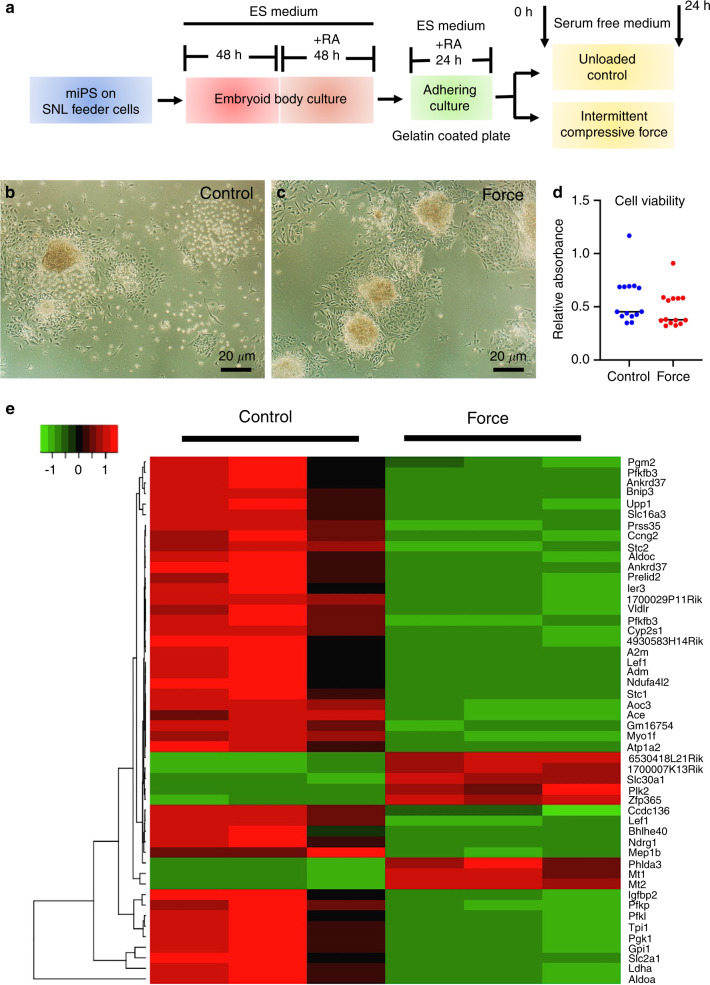
Differential gene expression profiles of intermittent compressive force (ICF)-treated iPSCs. The experimental scheme is shown in (**a**). Mouse induced pluripotent stem cells (iPSCs) were treated with ICF in serum-free conditions for 24 h. Cell viability was investigated using the MTT assay (**b**–**d**). RNA sequencing analysis was performed. Heat map shows the top 50 significant differentially regulated genes (**e**)

**Fig. 2:**
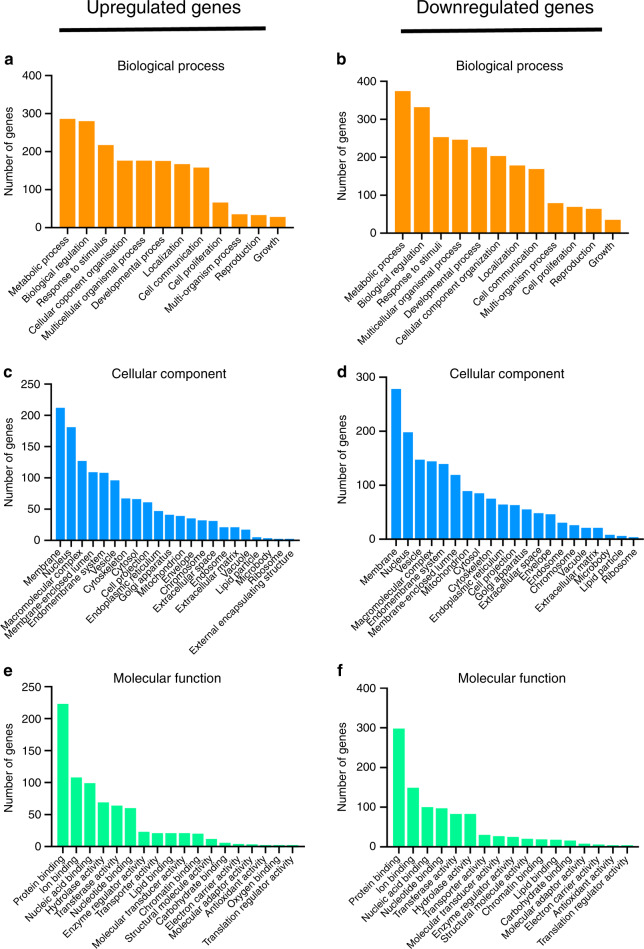
Gene ontology analyses of the upregulated and downregulated genes. The differentially regulated genes were mainly associated with metabolic process, membrane, and protein binding in the categories of biological process (**a**, **b**), cellular component (**c**, **d**), and molecular function (**e**, **f**), respectively

To examine the pathways regulated by ICF in iPSCs in a 3D form, embryoid bodies formed from mouse iPSCs were plated on a tissue culture plates. During embryoid body formation, the cells were cultured in the presence of retinoic acid to form relatively homogeneous immature mesenchymal cell constructs.^17^ iPSC constructs attached on the plates were then treated with ICF in serum-free medium for 24 h, followed by total RNA isolation for high-throughput RNA sequencing (Fig. 1a). After 24 h of ICF treatment, there was no significant morphological change in the iPSC constructs compared to the unloaded control (Fig. 1b, c). ICF did not affect cell viability at 24 h compared with the unloaded control (Fig. 1d). ICF treatment upregulated 893 genes and downregulated 1,076 genes. The top 50 differentially regulated (upregulated and downregulated) genes are shown in Fig. 1e and the top 20 significantly upregulated and downregulated genes are shown in Table 1. Over-representation analysis was performed and the number of genes in each gene ontology analysis for the upregulated and downregulated genes are illustrated in Fig. 2a–f. The differentially regulated genes were mainly associated with metabolic process, membrane, and protein binding in the categories of biological process, cellular component, and molecular function, respectively.

**Table 1:** Top 20 significantly upregulated and downregulated genes in the ICF-treated mouse iPSCs

Items	Gene symbol	Gene name	Log2FC	FDR
Upregulated genes	*Mt2*	Metallothionein 2	1.679 881 12	1.92E-57
	*Zfp365*	Zinc finger protein 365	1.414 175 91	1.92E-41
	*Mt1*	Metallothionein 1	1.379 982 17	4.92E-35
	*Fam212b*	Inka box actin regulator 2	1.780 233 32	3.48E-32
	*Slc30a1*	Solute carrier family 30 (zinc transporter), member 1	1.095 122 89	2.64E-19
	*1700007K13Rik*	RIKEN cDNA 1700007K13 gene	1.055 539 59	1.68E-18
	*Phlda3*	Pleckstrin homology-like domain, family A, member 3	0.920 961 22	1.09E-17
	*Mapkapk3*	Mitogen-activated protein kinase-activated protein kinase 3	0.761 985 97	1.11E-17
	*Btg2*	B cell translocation gene 2, anti-proliferative	0.700 049	1.50E-16
	*Celf5*	CUGBP, Elav-like family member 5	0.844 8561 7	1.32E-14
	*Cpt1c*	Carnitine palmitoyltransferase 1c	0.636 578 96	3.96E-14
	*Nefh*	Neurofilament, heavy polypeptide	0.858 813 64	4.43E-14
	*Plk2*	Polo-like kinase 2	1.017 012 41	6.14E-14
	*Gas6*	Growth arrest specific 6	0.602 381 47	1.24E-13
	*Mcam*	Melanoma cell adhesion molecule	0.904 323 58	3.62E-12
	*Eng*	Endoglin	0.604 656 63	4.82E-12
	*Ccng1*	Cyclin G1	0.878 373 39	1.06E-11
	*Zscan10*	Zinc finger protein 365	0.575 425 24	1.25E-11
	*Sesn2*	Sestrin 2	0.521 310 1	2.36E-11
	*Sytl1*	Synaptotagmin-like 1	1.102 422 23	4.50E-11
Downregulated genes	*Bnip3*	BCL2/adenovirus E1B interacting protein 3	−2.220 347	1.19E-74
	*Pfkp*	Phosphoglycerate kinase 1	−1.173 744	2.75E-64
	*Slc16a3*	Solute carrier family 16 (momocarboxylic acid transporters), member 3	−2.000 059 7	9.23E-59
	*A2m*	Alpha-2-macroglobulin	−2.577 263 7	4.06E-46
	*Ankrd37*	Ankyrin repeat domain 37	−2.275 651 2	4.72E-46
	*Ldha*	Lactate dehydrogenase A	−1.538 845 6	6.06E-44
	*Pgk1*	Phosphoglycerate kinase 1	−1.366 441 4	7.39E-37
	*Aldoc*	Aldolase C, Fructose-bisphosphate	−1.896 344 9	1.16E-35
	*Adm*	Adrenomedullin	−2.304 846 3	3.42E-35
	*Aoc3*	Amine oxidase, copper containing 3	−2.208 889 8	2.51E-34
	*Upp1*	Uridine phosphorylase 1	−1.347 068 4	2.92E-34
	*Tpi1*	Triosephosphate isomerase 1	−1.246 284 9	7.86E-33
	*Vldlr*	Very low density lipoprotein receptor	−1.122 410 4	4.69E-28
	*Aldoa*	Aldolase A, Fructose-bisphosphate	−1.171 554 2	8.41E-28
	*Ccng2*	Cyclin G2	−1.030 579 6	2.81E-27
	*Lef1*	Lymphoid enhancer binding factor 1	−1.395 389 8	1.48E-26
	*1700029P11Rik*	Riken cDNA 1700029P11 gene	−1.043 410 5	8.56E-26
	*Grin1*	Glutamate receptor, ionotropic, NMDA1 (zeta1)	−1.880 521 9	1.84E-25
	*Me1*	Malic enzyme 1, NADP(+)-dependent, cytosolic	−0.767 628 1	2.15E-25
	*Slc2a1*	Solute carrier family 2 (facilitated glucose transporter), member 1	−1.269 661 4	1.88E-24

A network topology-based analysis according to the protein-protein interaction network BIOGRID functional database was performed to enrich the gene ontology in the biological process category. The top 10 enriched gene ontology categories for up- and downregulated genes are shown in Fig. S1a and S1b. The enriched gene ontology categories for the upregulated genes were related to cell cycle (Fig. S1a), whereas the enriched gene ontology categories for the downregulated genes were related to small molecule and cofactor metabolic processes (Fig. S1b).

### ICF affected the p53 and cell cycle pathways in iPSCs

**Fig. 3:**
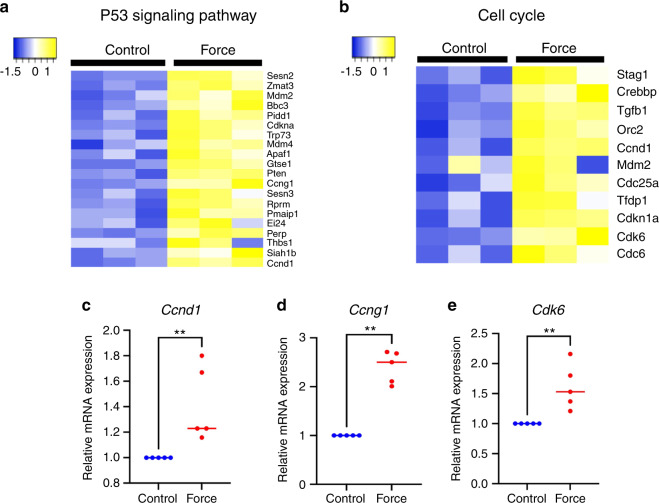
ICF regulates the P53 and cell cycle pathways in iPSCs. RNA sequencing analysis of iPSCs treated with ICF and control was performed using the Kyoto Encyclopedia. Heat map illustrates differentially expressed genes in the P53 signaling pathway and cell cycle pathways (**a**, **b**). Graph represents the mRNA expression of selected genes (*Ccnd1, Ccng1*, and *Cdk6*) from RNA sequencing results (**c**–**e**). Bars indicate significant difference between conditions (**P < 0.01)

Bioinformatic analysis using the Kyoto Encyclopedia of Genes and Genomes (KEGG) database revealed several pathways regulated by ICF. The upregulated genes were involved in the p53 signaling and FoxO signaling pathways, whereas the downregulated genes were involved in the HIF-1 signaling pathway (Table 2). Twenty genes in the p53 signaling pathway (Fig. 3a) and 11 genes in cell cycle pathways (Fig. 3b) were differentially expressed with ICF treatment compared to the control. All 20 differentially expressed genes shown in Fig. 3a were distributed in the pathway map of the KEGG database for the p53 signaling pathway (map04115) (Fig. S2), indicating that ICF affected most arms of the p53 pathway, including cell cycle (G1 arrest), apoptosis, DNA repair/damage prevention and p53 negative feedback. Ccnd1, which forms a complex with Cdk6 to regulate G1/S transition, was present in the heat maps for both the p53 signaling pathway and cell cycle (Fig. 3a, b). Cyclin G1 (Ccng1) is upregulated after DNA damage and functions as a negative feedback system that attenuates the activity of p53.^18^ Based on these results, we focused on Ccnd1, Cdk6, and Ccng1 to validate the mRNA expression of these genes using real-time quantitative polymerase chain reaction (PCR) (Fig. 3c–e). ICF induced *Ccnd1, Ccng1*, and *Cdk6* mRNA expression in iPSCs similarly to the results of the RNA sequencing analysis (Fig. 3c–e).

**Table 2:** Top 10 KEGG enriched pathways for upregulated and downregulated genes in ICF-treated mouse iPSCs

Items	ID	Name	Number of Genes	FDR
Upregulated pathway	mmu04115	p53 signaling pathway-Mus musculus (mouse)	22	2.95e-12
	mmu05206	MicroRNAs in cancer-Mus musculus (mouse)	19	6.04e-04
	mmu03460	Fanconi anemia pathway-Mus musculus (mouse)	8	9.7e-02
	mmu05218	Melanoma-Mus musculus (mouse)	9	1.69e-01
	mmu01524	Platinum drug resistance-Mus musculus (mouse)	9	2.3e-01
	mmu04110	Cell cycle-Mus musculus (mouse)	12	2.3e-01
	mmu03440	Homologous recombination-Mus musculus (mouse)	6	2.54e-10
	mmu04068	FoxO signaling pathway-Mus musculus (mouse)	12	3.18e-01
	mmu05215	Prostate cancer-Mus musculus (mouse)	9	3.34e-01
	mmu00514	Other types of O-glycan biosynthesis-Mus musculus (mouse)	4	3.34e-01
Downregulated pathway	mmu01230	Biosynthesis of amino acids -Mus musculus (mouse)	25	5.48e-11
	mmu01200	Carbon metabolism-Mus musculus (mouse)	30	6.42e-11
	mmu01100	Metabolic pathways-Mus musculus (mouse)	130	6.42e-11
	mmu00010	Glycolysis/gluconeogenesis-Mus musculus (mouse)	19	9.15e-08
	mmu00100	Steroid biosynthesis-Mus musculus (mouse)	10	8.04e-07
	mmu00030	Pentose phosphate pathway-Mus musculus (mouse)	11	2.74e-05
	mmu00500	Starch and sucrose metabolism-Mus musculus (mouse)	11	3.36e-05
	mmu04066	HIF-1 signaling pathway-Mus musculus (mouse)	20	5.81e-05
	mmu03010	Ribosome-Mus musculus (mouse)	22	2.56e-04
	mmu00520	Amino sugar and nucleotide sugar metabolism -Mus musculus (mouse)	12	2.81e-04

**Fig. 4:**
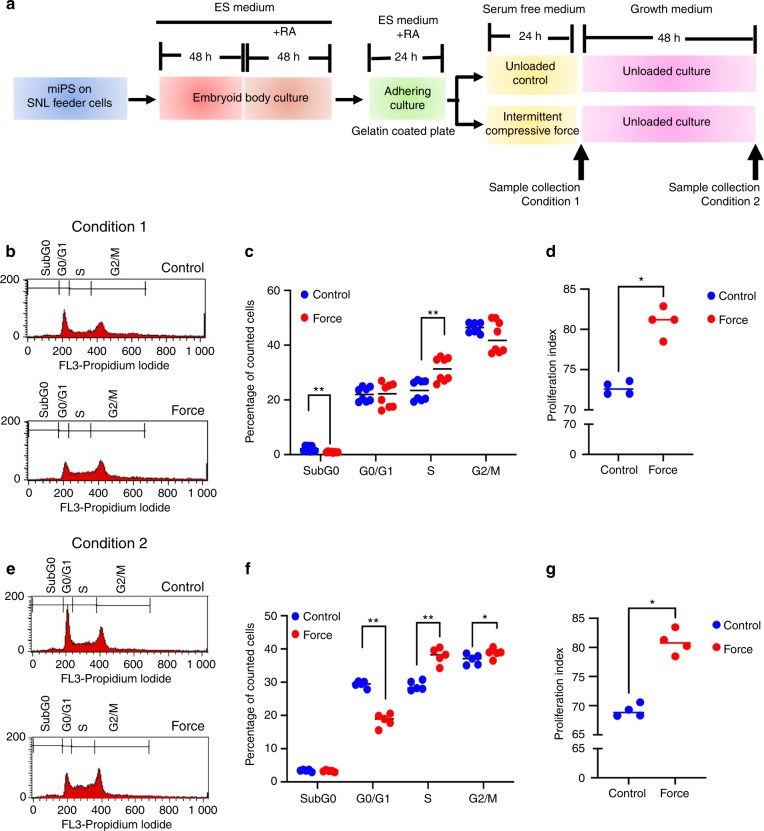
ICF promotes cell cycle progression. The experimental scheme is illustrated in (**a**). Cell cycle analysis of conditions: (1) iPSCs were treated with ICF for 24 h in serum-free medium (**b**, **c**), and (2) 24 h after loading, the cells were maintained in normal growth medium for 48 h (**e**, **f**). The proliferative index in conditions 1 and 2 is shown in (**d**, **g**). Bars indicate a significant difference between conditions (*P < 0.05. **P < 0.01)

To further confirm the biological function, cell cycle analysis using flow cytometry was performed after ICF treatment for 24 h in serum-free medium (condition 1). ICF treatment resulted in a reduction of the SubG0 population and increase in the S phase population (Fig. 4b, c). After ICF, cells were maintained for 48 h in normal growth medium (in the presence of serum) (condition 2) and used for the cell cycle analysis (Fig. 4a). The G0/G1 population was significantly decreased, whereas the S and G2/M populations were markedly increased after ICF stimulation compared with the control (Fig. 4e, f). The proliferative index was significantly increased in both conditions (Fig. 4d, g).

**Fig. 5:**
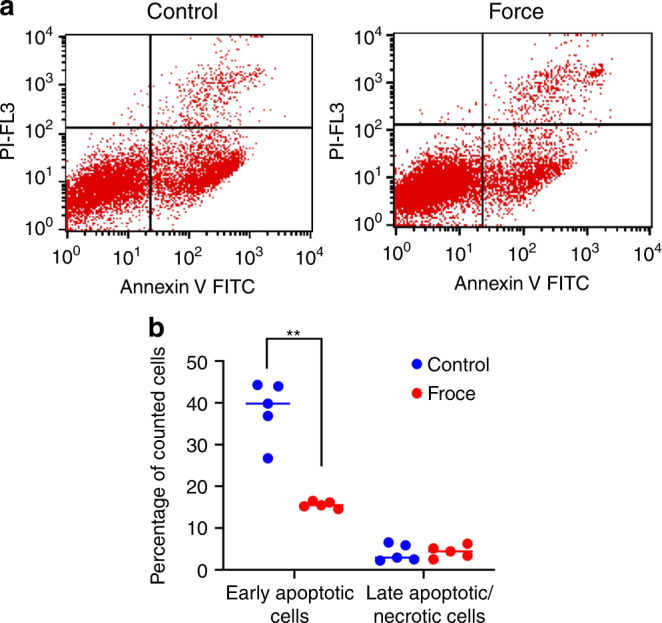
ICF reduces the number of early apoptotic cells. iPSCs were treated with ICF for 24 h in serum-free medium. Cells were stained with Annexin V and propidium iodide (PI). The percentage of early and late apoptotic cells is shown in (**a**, **b**). Bars indicate a significant difference between conditions (**P < 0.01)

The reduction of the SubG0 population with ICF treatment implied a reduction of cell apoptosis. To confirm the reduction of apoptosis, the cells were fluorescently stained with annexin V and propidium iodide (PI) to identify cells in early and late apoptosis. Flow cytometry showed that ICF significantly attenuated the number of early apoptotic cells but did not affect the number of late apoptotic cells (Fig. 5a, b).

### ICF modulated the expression of cell cycle-related genes via TGF-β signaling

**Fig. 6:**
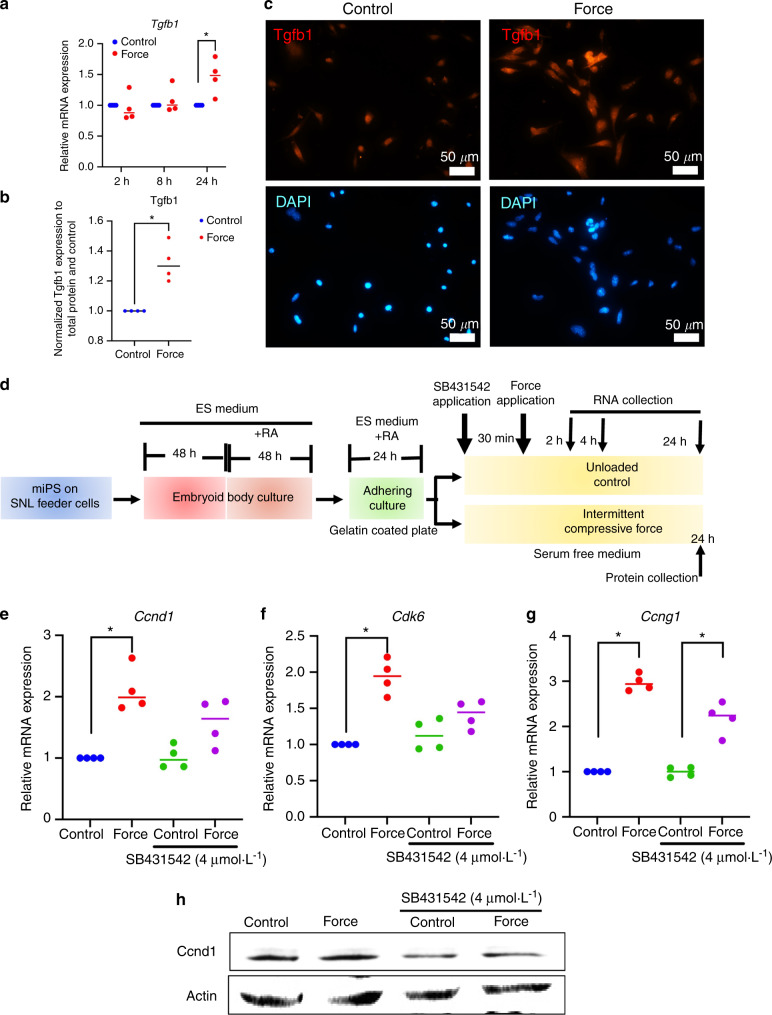
ICF regulated cell cycle-related gene expression via the TGF-β signaling pathway. iPSCs were treated with ICF in serum-free medium. *Tgfb1* mRNA expression was investigated using real-time PCR at 2, 8, and 24 h (**a**) and Tgfb1 protein expression was determined by ELISA (**b**) and immunofluorescence staining (**c**) at 24 h. DAPI was used for nuclear staining. Scale bars: 50 μm. In the TGF-β inhibition experiment, cells were treated with SB431542 (4 μmol·L^−1^) for 30 min prior to exposure to the force. The experimental scheme is illustrated in (**d**). The mRNA of *Ccnd1, Ccng1*, and *Cdk6* is shown in (**e**–**g**). The protein expression of Ccnd1 was examined using western blot analysis (**h**). Bars indicate a significant difference between conditions (*P < 0.05, **P < 0.01)

TGF-β signaling is well known to be stimulated in stem cells by mechanical stresses.^10,19^ Because Tgfb1 was identified as a cell cycle gene differentially expressed by ICF stimulation (Fig. 3b line 3), we focused on TGF-β signaling as a possible mechanism underlying modulation of cell cycle-related gene expression by ICF. Real-time quantitative PCR analysis revealed that ICF stimulation significantly upregulated *Tgfb1* mRNA expression after 24 h (Fig. 6a). The significant Tgfb1 protein expression at 24 h was also confirmed by enzyme-linked immunosorbent assay (ELISA) and immunofluorescence staining (Fig. 6b, c). To investigate the involvement of TGF-β signaling in the modulation of the cell cycle by ICF, iPSCs were pretreated with 4 μmol·L^−^^1^ SB431542, an inhibitor of the TGF-β receptor, for 30 min prior to ICF application (the experimental scheme is illustrated in Fig. 6d). SB431542 attenuated the effect of ICF on *Ccnd1* and *Cdk6* mRNA expression (Fig. 6e, f). In contrast, *Ccng1* mRNA expression levels were not significantly changed with SB431542 pretreatment (Fig. 6g). Corresponding with the mRNA results, SB431542 pretreatment decreased force-induced Ccnd1 protein expression (Fig. 6h).

## Discussion

The present study investigated the effect of ICF on the gene expression profile of iPSCs in a 3D construct form. Gene ontology analyses demonstrated ICF-induced differential expression of genes mainly associated with metabolic process, biological function, and response to stimulus as biological process categories; membrane and nucleus as cellular component categories; and protein binding, ion binding, and nucleic acid binding as molecular function categories. These results suggest that iPSCs indeed sensed mechanical stimulation by ICF to regulate a variety of molecular mechanisms that resulted in several biological effects, such as metabolic control of the cell cycle. The cellular response to ICF seems to depend on stem cell type because our previous study using the same ICF assay on PDLCs showed significant differences in differentially regulated genes mainly associated with calcium signaling, focal adhesion, and TGF-β pathways.^10,14^ Although the specificity of the gene expression profile induced by ICF was not determined in this study, the type of compressive force should have a specific influence on the gene expression profile because a previous report showed that static compressive force and ICF differentially regulated gene expression in PDLCs.^10^ Not only mRNA but also miRNA and long non-coding RNA are also regulated by mechanical force. Application of tensile force to PDLCs regulates the expression of many miRNAs, and the pathways enriched in tensile force-treated cells include many signaling pathways including MAPK, Rap1, and Hippo.^20^ Static compressive force treatment of PDLCs results in a change in long non-coding RNA expression in many gene ontologies including extracellular matrix (ECM) organization, collagen fibril organization, and cellular response to hypoxia.^21^ Hence, it is important to investigate the effect of specific mechanical treatment conditions on specific cell types using multiple omics analyses.

In this study, the network topology-based analysis for the protein-protein interaction network demonstrated that ICF induced genes in cell cycle categories in iPSCs, whereas genes involved in metabolic processes were downregulated. Correspondingly, a systematic review of gene expression in PDLCs previously illustrated that static compressive force regulated numerous enriched pathways including ECM organization, canonical glycolysis, glycolytic process, cell adhesion, cell-matrix adhesion, integrin-mediated signaling, and cell adhesion mediated by integrin in two-dimensional (2D) culture.^22^ In contrast, compressive loading in 3D-cultured PDLCs was shown to regulate pathways related to cell cycle, cell division, cell proliferation, and mitotic process-related pathways.^22^ In the present study, ICF was applied to iPSCs in 3D form and found to mainly induce genes for the cell cycle. This implies that 3D cell constructs might be susceptible to cell cycle induction by compressive forces, which may have implications for manipulating iPSCs for in vitro organoid formation.

The role of mechanical stimulation in cell growth is still controversial. Application of high pressure to Wharton jelly-derived MSCs under hypoxic conditions has been shown to promote cell proliferation.^23^ The magnitude of the pressure also influenced this effect, as a pressure of 2.0 PSI induced more cell proliferation than 2.5 PSI.^23^ In human embryonic lung fibroblasts, the effect of mechanical stretch on cell proliferation was shown to be magnitude-specific.^24^ Specifically, 5% and 10% stretching resulted in an increase in the proliferation index, whereas a higher magnitude of stretching (15% and 20% elongation) led to reduced proliferation.^24^ Static compressive force was shown to increase the proliferation index in human gingival fibroblasts and this effect was attenuated by inhibition of the TGF-β receptor.^25^ On the contrary, the static compressive force of 2 g·cm^−1^ decreased PDLC viability and proliferation.^26^ Specifically, the number of trypan blue-positive cells increased over time upon static compressive force treatment.^26^ Another study employing the same force type and magnitude for PDLCs reported that static compressive force reduced cell proliferation and the Ki67-positive cell population.^27^ This phenomenon occurred via regulation of *MIR31HG*.^27^

The present study found that ICF treatment for 24 h in the serum-free medium led to a significant increase in the proliferation index. In addition, cells pretreated with ICF for 24 h and subsequently maintained in a normal growth medium showed a significant increase in proliferation index. These results indicate that ICF promotes the growth of iPSCs, which is consistent with the gene expression profile analysis. In addition, we observed that ICF increased the percentage of cells in S phase. This is consistent with previous studies showing that uniaxial compressive stress increased the percentage of cells in the S and G2/M phases over time in human dental pulp cells.^28^ Cyclic tensile strain has also been shown to increase the G2/M and S phase populations in rat growth plate chondrocytes, and this stimulatory effect was attenuated when cells were treated with YAP and ERK inhibitors.^29^ It has also been shown that nuclear envelope flattening by compressive force promotes the transition from G1 to S phase in HeLa cells.^30^

However, there are also reports indicating negative effects of mechanical force on cell cycle progression. For example, laminar shear stress was shown to reduce [3H]thymidine incorporation over time.^31^ This reduction corresponded with an increase in the G0/G1 population, indicating that laminar shear stress-induced cell cycle arrest in rat bone marrow-derived MSCs.^31^ Similarly, in smooth muscle cells, cyclic stretching led to accumulation of cells in the G0/G1 phase, which was associated with reduced retinoblastoma protein phosphorylation and increased p21 levels.^32^ Correspondingly, the expression of the cell cycle markers MCM2, cyclin A, PCNA, and cyclin D decreased with static compressive force treatment.^26^ Many factors can explain the discrepant findings regarding the effects of mechanical force on cell cycle progression, for example, different cell types, force parameters, and culture conditions. Nonetheless, a generalized understanding of the effects would facilitate the control of cell-cell interactions for engineered cell manipulation.

In the present study, ICF attenuated the Sub G0 population, corresponding with a significant decrease in the percentage of cells in the early apoptotic phase (annexin V^+^/PI^**−**^ cells). Similarly, static force application in a previous study increased the annexin V^+^/7AAD^**−**^ and annexin V^+^/7AAD^+^ cell populations in human PDLCs over time.^26^ Laminar shear stress reduces the proportion of early apoptotic cells and induced *Bcl-2* in rat bone marrow-derived MSCs, with a greater effect observed for higher magnitudes of stress.^31^ Hydrostatic compressive force decreases caspase-3 expression in fibrochondrocytes, leading to inhibition of cell apoptosis.^33^ This effect involves signal transduction from integrin α5 and β1 receptors.^33^ Similarly, hydrostatic compressive force reduced chondrocyte apoptosis, with a greater effect observed for higher magnitudes of stress, and this effect occurred through the integrin-FAK-ERK/PI3K pathway.^34^ Our data, together with these reports, appear to contradict the general idea that exposure to cellular stress stimulates p53 to induce apoptosis.^35^ This discrepancy may be explained by the fact that cellular responses through the p53 pathway are influenced by several factors, including the type of stress and cells as well as the activity of p53 co-activators.^35^ It is known that Ccng1 expression, which can be regulated by p53 in response to DNA damage^36^, functions together with murine double minute 2 (Mdm2) to decrease p53 activity in a negative feedback loop.^18^ In contrast, without Ccng1, Mdm2 inhibits p53 activity by ubiquitinating p53 through the E3 ligase activity, blocking p53 from binding to its target transcription sites and exporting p53 from the nucleus.^37^ In the present study, both Ccng1 and Mdm2 were differentially expressed in iPSCs in response to ICF (Fig. 3a), and this was reflected in the “p53 negative feedback” pathway identified in the KEGG database (Fig. S2). We speculate that the iPSCs upregulated Ccng1 and Mdm2 in response to ICF, that might, in turn, activate the p53 negative feedback pathway to reduce apoptosis. The mechanism of how ICF reduces iPSC apoptosis should be further investigated in detail.

TGF-β signaling has been shown to participate in the regulation of cell behavior by mechanical force. Our previous studies showed that ICF increased sclerostin and periostin expression via the TGF-β pathway in human PDLCs to maintain periodontal tissue homeostasis and regeneration.^38^ In addition, pretreatment of PDLCs with ICF promoted osteogenic differentiation via TGF-β signaling.^10^ Mechanical force generated by shaking culture-induced chondrogenic differentiation via TGF-β and Wnt signaling in 3D iPSC constructs.^19^ Tensile force upregulated scleraxis, inhibiting ossification in PDLCs through TGF-β-ephrin A2 signaling.^39^ In human gingival fibroblasts, compressive force increased cell proliferation, ECM synthesis and TGF-β expression, and a TGF-β inhibitor attenuated these effects.^25^ In this study, ICF significantly induced mRNA and protein expression of Tgfb1 in iPSCs. Pretreatment with a TGF-β inhibitor (SB431542) prior to force application attenuated ICF-induced Ccnd1 and *Cdk6* expression. Correspondingly, knockdown of latent TGF-β binding protein 1 decreased expression of cyclinD1, CDK4 and Ki-67, and inhibited cell cycle progression in natural killer/T cell lymphoma cells, suggesting a role for TGF-β signaling in cell proliferation and cell cycle progression.^40^ Conversely, a study in human endometrial cancer cells showed that withaferin A, an antiproliferative drug used in cancer treatment, suppressed proliferation, cell cycle, migration, and invasion and decreased TGF-β-related protein expression, implying that TGF-β signaling is involved in the inhibition of proliferation.^41^ In this study, the KEGG database indicated that Tgfb1 was differentially regulated in relation to the cell cycle in response to ICF (Fig. 3b). To our best knowledge, this is the first study to demonstrate the role of TGF-β signaling in enhancing cellular proliferation and decreasing apoptosis in iPSCs in response to ICF, which could contribute to a protocol to manipulate iPSCs for organoid fabrication.

## Conclusion

ICF promoted iPSC proliferation and cell cycle and suppressed apoptosis. TGF-β signaling may be involved in ICF-induced cell cycle progression in iPSCs.

## Materials and methods

### iPSC culture and preparation

Mouse gingival fibroblast-derived iPSCs^42^ were cultured as described in previous reports.^43,44^ Briefly, cells were seeded on SNL feeder cells in ESC culture medium. The medium was Dulbecco’s modified Eagle medium (Gibco, Grand Island, NY, USA) containing 15% fetal bovine serum (Gibco), 2 mol·L^−1^ L-glutamine (GlutaMAX-1, Gibco), 100 units per mL penicillin, 100 μg·mL^−1^ streptomycin (EmbryoMax, Millipore, Temecula, CA, USA), 0.1 mmol·L^−1^ non-essential amino acid (Sigma-Aldrich, St. Louis, MO, USA), and 0.1 mmol·L^−1^ 2-mercaptoethanol (Gibco).

For embryoid body formation, iPSCs were selectively trypsinized to remove SNL feeder cells and seeded on Corning Elplasia 24-well round-bottom plates, which contain hundreds of ultra-low attachment surface microwell spots per well (Corning, Oneonta, NY, USA), to fabricate embryoid bodies with equivalent size.^45^ Then, iPSCs were maintained for 2 days in ESC culture medium. Subsequently, the embryoid bodies were maintained in ESC culture medium supplemented with 1 μmol·L^−^^1^ all-trans retinoic acid (Sigma-Aldrich) for 2 days to guide differentiation into immature mesenchymal cells.^17^ iPSC embryoid bodies were then transferred to 0.1% gelatin-coated tissue culture plates at 250 000 cells per cm^2^ and maintained for 1 day in the same medium before mechanical force stimulation. A schematic diagram for the cell culture is shown in Fig. 1a.

### ICF treatment

ICF was applied using a computer-controlled apparatus.^10^ Retinoic acid-treated iPSCs were seeded on 0.1% gelatin-coated 6-well tissue culture plates (Corning) for 1 day. Subsequently, ICF was applied with 1.5 g·cm^−2^ force and 0.23 Hz frequency for 24 h in SFM.^10^

### RNA sequencing

After mechanical stimulation for 24 h, total cellular RNA was extracted using Ribospin II (GeneAll, Seoul, South Korea). Three independent experiments were performed. RNA quality and quantity were first screened using Nanodrop and further assessed using an Agilent 2100 BioAnalyzer (Agilent Technologies, CA, USA) as well as a Qubit RNA HS assay kit (ThermoFisher Scientific, MA, USA). The library was prepared using a NEBNext Ultra Directional RNA Library Prep Kit for Illumina (New England Biolabs, MA, USA) to obtain a strand-specific mRNA library. Single-ended sequencing was performed using an Illumina Nextseq platform with 75 cycles. Read quality was checked and low-quality reads were filtered using FastQC and Trimmomaic, respectively.^46^ Processed reads were mapped to a reference genome, G, and sorted by creating a sorted bam file using HISAT2 and Samtools.^47,48^ Gene transcripts of each sample were counted using HTSeq.^49^ Differential gene expression was examined using DEseq2.^50^ A significant difference was established when the false discovery rate was less than 0.05. Raw sequencing and read count data were deposited in the NCBI Sequence Read Archive (SRP158658) and NCBI Gene Expression Omnibus (GSE118952). Gene ontology and enriched pathways were analyzed using WebGestalt and Reactome.^51,52^ The KEGG database was also employed as a reference.^53^ Heatmap was generated using Heatmapper.^54^

### Real-time quantitative PCR

One microgram of the total cellular RNA was converted to complimentary DNA using a reverse transcription kit (ImProm-II, Promega, Madison, WI, USA). Subsequently, the complimentary DNA was further analyzed via polymerase chain reaction using a SYBR Green detection system (FastStart Essential DNA Green Master kit, Roche Diagnostics, Mannheim, Germany) on a LightCycler 96 real-time PCR system (Roche Diagnostics). The amplification profile was 40 cycles of denaturation at 95 °C, annealing at 60 °C, and extension at 72 °C for 20 s of each step. *Gapdh* was used as the reference gene. Primer sequences are shown in Table S1.

### MTT assay

Cells were exposed to ICF for 24 h. The culture medium was replaced with MTT solution (0.5 mg·mL^−1^) and incubated for 30 min. The precipitated formazan crystals were dissolved in DMSO/glycine buffer and the absorbance was determined at 570 nm using a microplate reader (Elx800, BIO-TEK, Winooski, VT, USA).

### Cell cycle analysis

After ICF treatment, the cells were fixed in cold 70% ethanol for 15 min and washed with cold PBS. A cell suspension was obtained in FACS buffer. RNase treatment was performed at room temperature for 30 min. Further, cells were stained with PI solution (Sigma-Aldrich) for 30 min at room temperature in the dark. The stained cells were evaluated using a FACSCalibur (BD Bioscience, San Jose, CA, USA). The proliferation index was calculated as follows; Proliferation index (%) = [(S + G2/M)/(G0/G1 + S + G2/M)] × 100%.

### Apoptosis assay

Cells were trypsinized and collected. The cell pellet was re-suspended in annexin-binding buffer (BioLegend, San Diego, CA, USA) and subsequently stained with annexin V-FITC (BioLegend) and PI for 10 min in the dark. The stained cells were examined using a FACSCalibur system.

### Immunofluorescence staining

Samples were fixed in 4% buffered formalin (Sigma-Aldrich) for 10 min at room temperature. The samples were then incubated with 0.1% Triton-X100 (USB corporation, Cleveland, OH, USA) for 5 min and subsequently immersed in 2% horse serum (Hyclone, South Logan, UT, USA) for 30 min at room temperature. The samples were stained with anti-Tgfb1 antibody (Abcam, Cambridge, UK) overnight at 4 °C. A biotinylated goat-anti rabbit antibody (Santa Cruz Biotechnology, Dallas, TX, USA) was used as a secondary antibody. Fluorescence labeling was performed with Streptavidin, Rhodamine Red-X conjugate (Invitrogen, MD, USA). Nuclei were stained with 4’, 6-diamidino-2-phenylindole (DAPI: Tocris Bioscience, Bristol, UK). The samples were then observed under an Apotome.2 (Carl Zeiss, Jena, Germany) fluorescence microscope.

### ELISA

Total protein was extracted from the cell lysate using RIPA buffer. Tgfb1 levels were examined using a human TGF-β1 immunoassay kit (DB100B, R&D Systems) according to the manufacturer’s instructions. The optical density was measured at 450 nm. Tgfb1 protein levels were calculated using a standard curve and further normalized to total protein and the control condition.

### Western blot analysis

Cellular proteins were extracted using RIPA buffer with a protease inhibitor cocktail (Sigma-Aldrich). The samples were electrophoresed on a 12% sodium dodecyl sulfate-polyacrylamide gel and further transferred onto nitrocellulose membranes. The membranes were incubated with primary antibody (rabbit anti-Ccnd1, Cell Signaling Technology; or anti-actin, Sigma-Aldrich) overnight. The secondary antibody was goat anti-rabbit antibody (sc-2040, Santa Cruz Biotechnology). The membranes were incubated with peroxidase-labeled streptavidin and the signal was examined using chemiluminescence (SuperSignal West Femto Maximum Sensitivity Substrate, ThermoFisher Scientific).

### Statistical analyses

Graphical illustration and statistical analyses were performed using Prism 8 (GraphPad Software, CA, USA). Data are shown in scatter plots and the line represents median values. Each dot represents an individual value. All experiments were performed in at least quadruplicate. Statistical analyses were performed using the Mann Whitney U test for two-group comparisons and the Kruskal Wallis test followed by pairwise comparisons for more than two-group comparisons. Significance was defined when *P* < 0.05.

## Supplementary information

Supporting Information

Supplementary Figure 1

Supplementary Figure 2
