# Supplementary material for: Intermittent compressive force induces cell cycling and reduces apoptosis in embryoid bodies of mouse induced pluripotent stem cells
Source: Int J Oral Sci. 2022 Jan 4;14:1. doi: 10.1038/s41368-021-00151-3 (PMC8724316; doi:10.1038/s41368-021-00151-3)
Supplement: Supplementary file 1 — Supporting Information [file 41368_2021_151_MOESM1_ESM.docx]

**Supporting Information**

**Intermittent compressive force induces cell cycling and reduces apoptosis in embryoid bodies of mouse induced pluripotent stem cells**

Jeeranan Manokawinchoke ^1,2^, Phoonsuk Limraksasin^1,2^, Hiroko Okawa^1^, Prasit Pavasant ^2^,

Hiroshi Egusa ^1,3,*^, Thanaphum Osathanon ^2,*^

^1^ Division of Molecular and Regenerative Prosthodontics, Tohoku University Graduate School of Dentistry, Sendai 980-8575, Japan

^2^ Dental Stem Cell Biology Research Unit and Department of Anatomy, Faculty of Dentistry, Chulalongkorn University, Bangkok, 10330 Thailand

^3^ Center for Advanced Stem Cell and Regenerative Research, Tohoku University Graduate School of Dentistry, Sendai, Miyagi 980-8575, Japan

**Running title:** Effects of intermittent compressive force on iPSCs

**Authors contact official email:**

Jeeranan Manokawinchoke: jeeranan.m@chula.ac.th

Phoonsuk Limraksasin: limraksasin.phoonsuk.b4@tohoku.ac.jp

Hiroko Okawa: hiroko.okawa.d3@tohoku.ac.jp

Prasit Pavasant: prasit.pav@chula.ac.th

Hiroshi Egusa: egu@tohoku.ac.jp

Thanaphum Osathanon: thanaphum.o@chula.ac.th

**Co-corresponding authors**

Hiroshi Egusa, DDS, PhD

Director, Center for Advanced Stem Cell and Regenerative Research

Professor and Chair, Division of Molecular and Regenerative Prosthodontics,

Tohoku University Graduate School of Dentistry

4-1 Seiryo-machi, Aoba-ku, Sendai-city, 980-8575, Japan

Tel: +81-22-717-8363, Fax: +81-22-717-8367

egu@tohoku.ac.jp

Thanaphum Osathanon, DDS, PhD

Professor, Dental Stem Cell Biology Research Unit, Faculty of Dentistry,

Chulalongkorn University,

34 Henri-Dunant Rd. Pathumwan, Bangkok, 10330 Thailand

Tel: +66-2-218-8885, Fax: +66-2-218-8870

Email: [thanaphum.o@chula.ac.th](mailto:thanaphum.o@chula.ac.th)

**
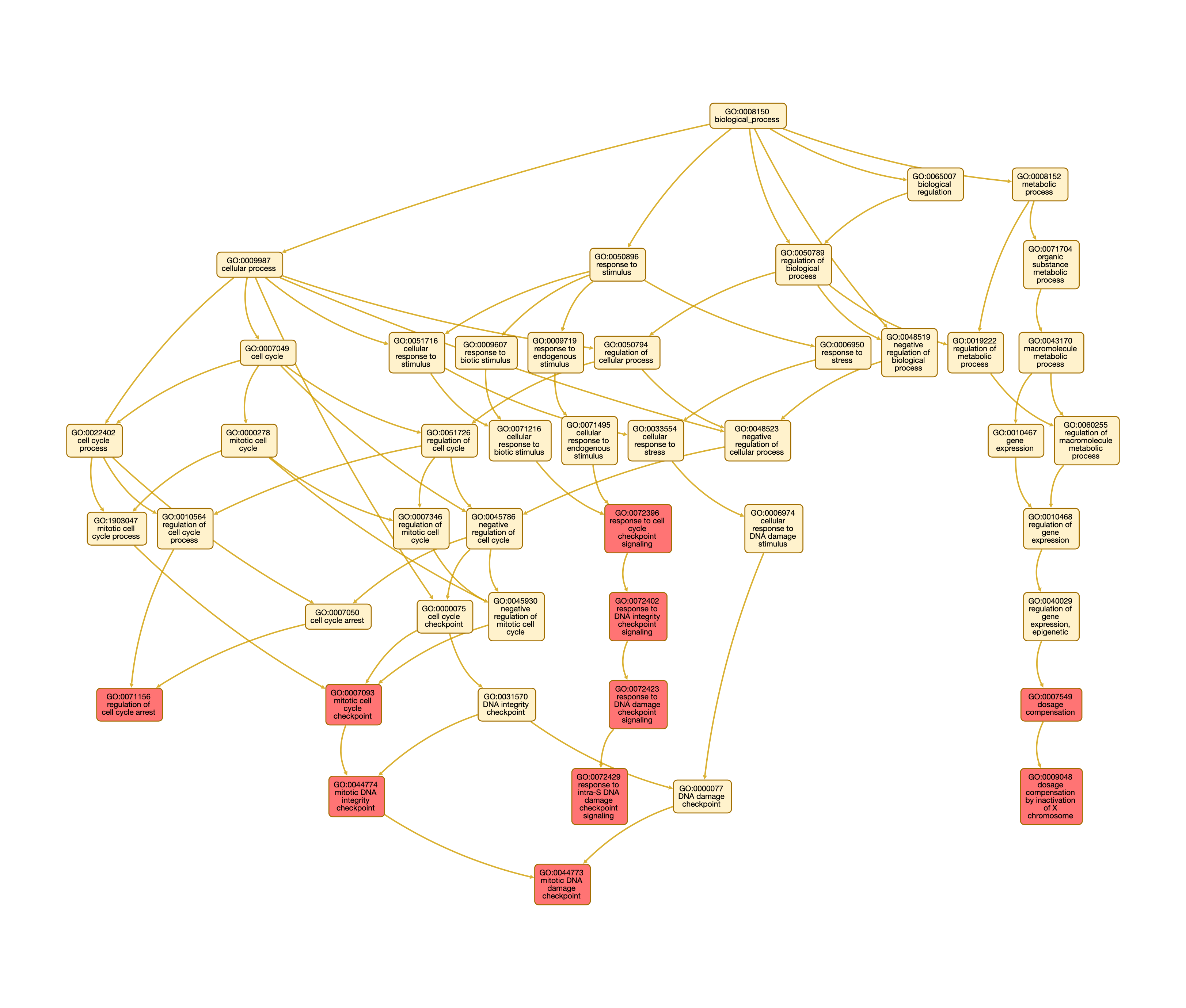
**

a

**
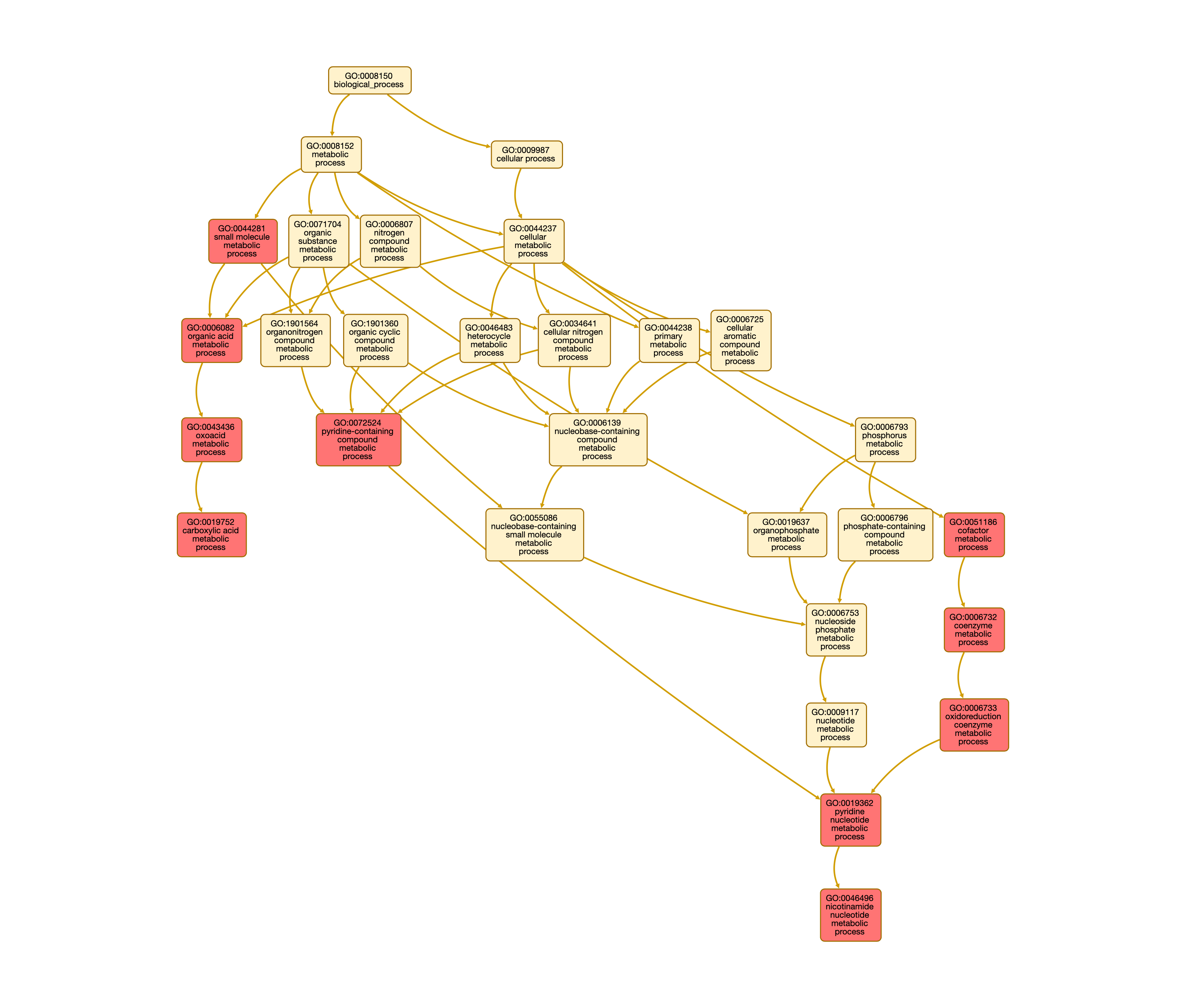
**

b

**Figure S1** Top 10 enriched gene ontology categories for up- and downregulated genes in ICF-treated iPSCs. A network topology-based analysis according to protein-protein interaction network BIOGRID functional database was performed. The top 10 enriched gene ontology categories for (a) up- and (b) downregulated genes are shown.


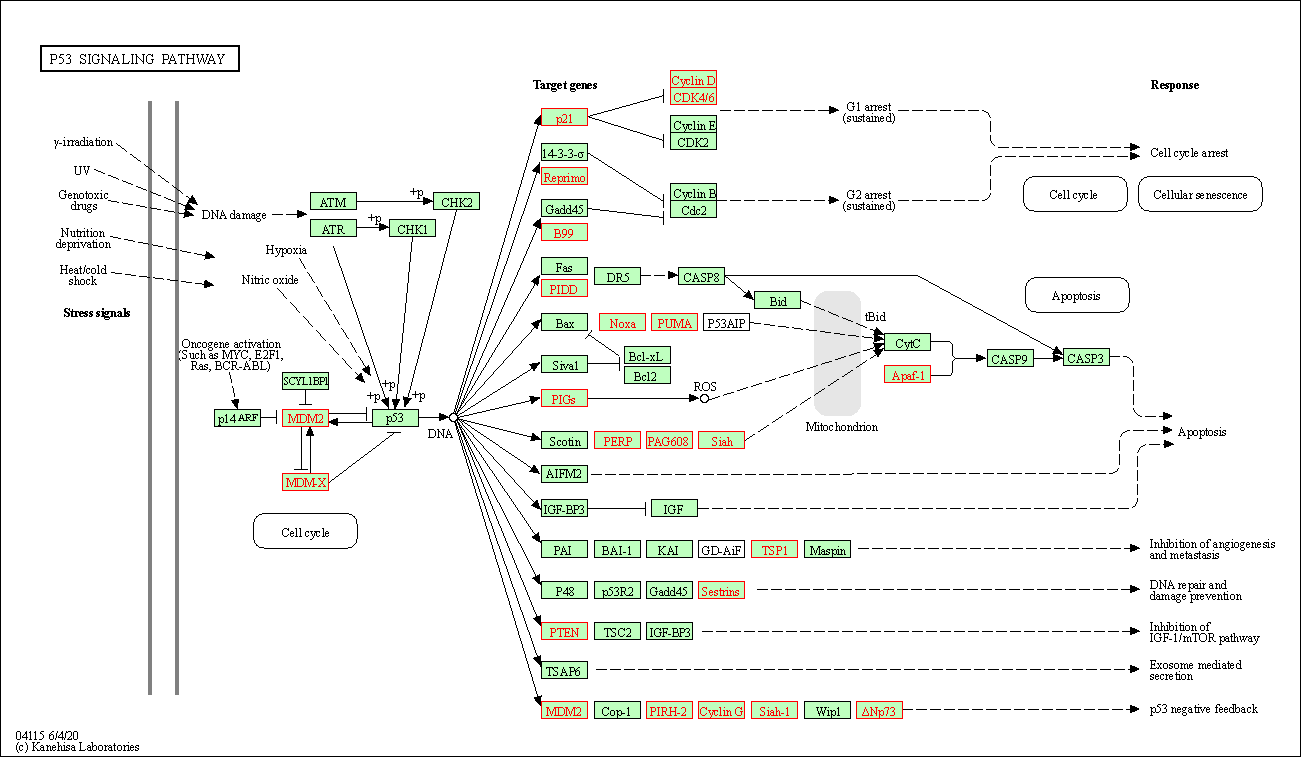


**Figure S2** KEGG diagram of differentially expressed genes in P53 signaling.

**Table S1.** Oligonucleotide sequences used in this study.

| **Gene** |  | **Primer sequences (F=forward, R=Reverse)** | **Accession number** | **Size (bp)** |
| --- | --- | --- | --- | --- |
| *Gapdh* | F | GGC AAA TTC AAC GGC ACA | NM_001289726.1 | 208 |
|  | R | CGG AGA TGA TGA CCC TTT |  |  |
| *Ccnd1* | F | GTT CAT TTC CAA CCC ACC CTC | NM_007631.2 | 117 |
|  | R | AGA AAG TGC GTT GTG CGG TAG |  |  |
| *Ccng1* | F | GCG TTG GAG ATC CAA GCA CTG A | NM_009831.2 | 113 |
|  | R | GGA AAC AAG CTC TTG CCA GAA GG |  |  |
| *Cdk6* | F | GGC GTA CCC ACA GAA ACC ATA | NM_009873.3 | 187 |
|  | R | AGG TAA GGG CCA TCT GAA AAC T |  |  |
