# Supplementary figures and images for: Intermittent compressive force induces cell cycling and reduces apoptosis in embryoid bodies of mouse induced pluripotent stem cells
Source: Int J Oral Sci. 2022 Jan 4;14:1. doi: 10.1038/s41368-021-00151-3 (PMC8724316; doi:10.1038/s41368-021-00151-3)

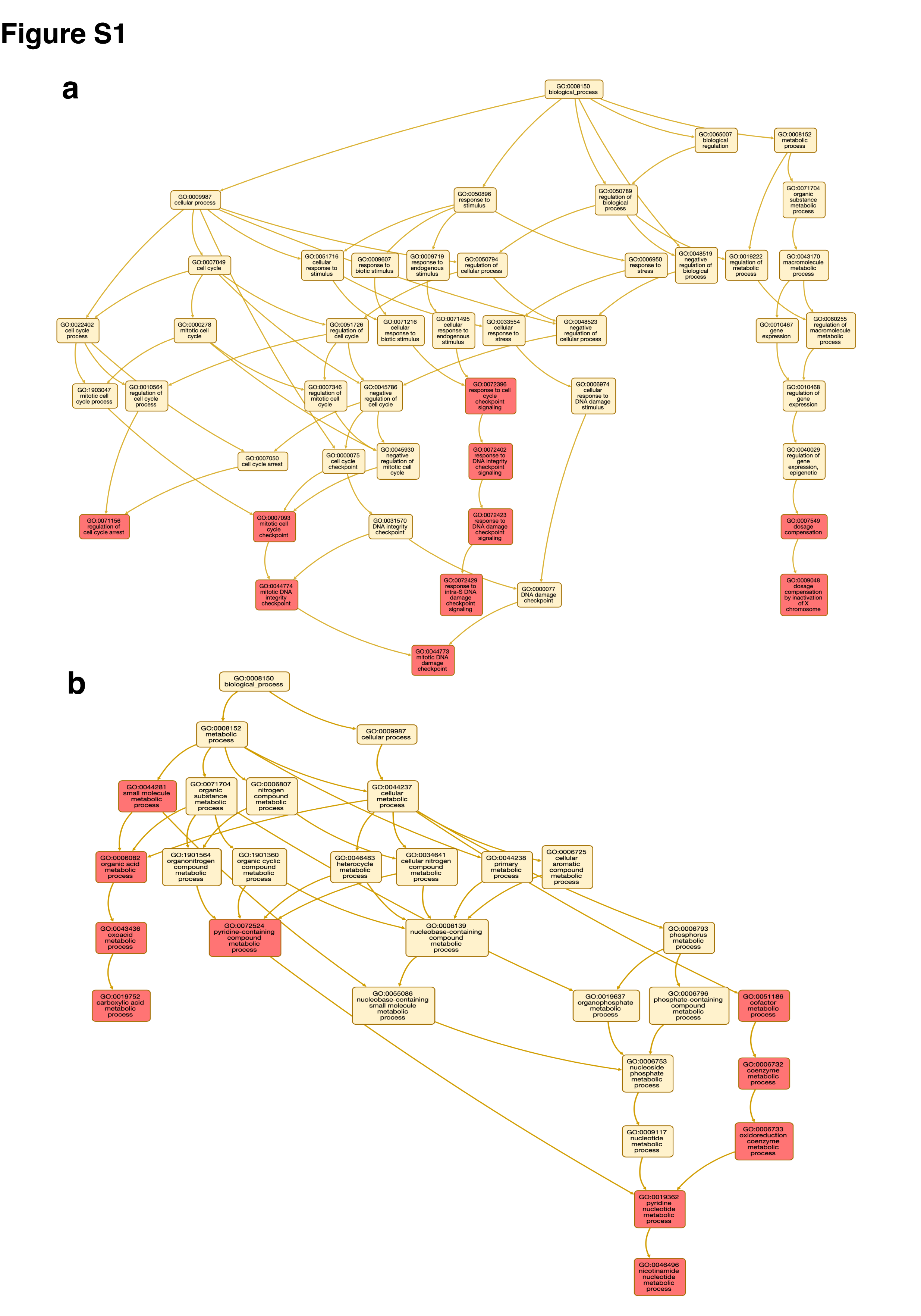

Supplement: Supplementary file 2 — Supplementary Figure 1 [file 41368_2021_151_MOESM2_ESM.tif]

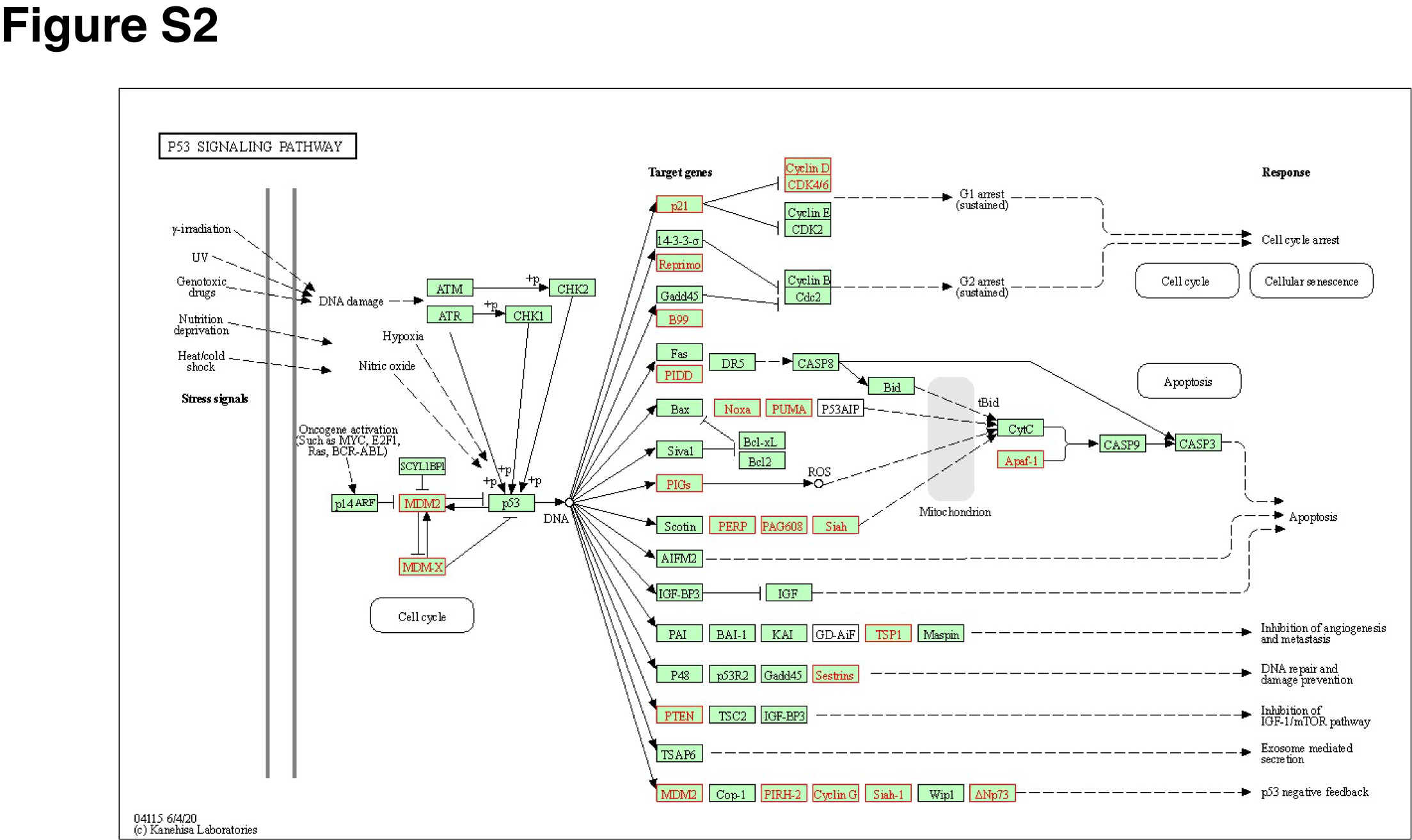

Supplement: Supplementary file 3 — Supplementary Figure 2 [file 41368_2021_151_MOESM3_ESM.tif]
